# Real-world practice patterns of eplerenone use for central serous chorioretinopathy

**DOI:** 10.1186/s40942-023-00500-w

**Published:** 2023-10-02

**Authors:** Ramesh Venkatesh, Vishma Prabhu, Aishwarya Joshi, Rubble Mangla, Rishi Singh, Lihteh Wu, Paolo Lanzetta, Baruch Kuppermann, Francesco Bandello, Francine Behar Cohen, Jay Chhablani

**Affiliations:** 1 Department of Retina and Vitreous, Narayana Nethralaya #121/C, 1st R block, Rajaji Nagar, Bangalore, 560022 India; 2 Cleveland Clinic Lerner College of Medicine, Cleveland, OH USA; 3 Cleveland Clinic Martin Health, Stuart, FL USA; 4 Macula, Vitreous and Retina Associates of Costa Rica, San José, Costa Rica; 5 Department of Medicine - Ophthalmology, University of Udine, Udine, Italy; 6 Istituto Europeo di Microchirurgia Oculare, Udine, Italy; 7 Department of Ophthalmology, Gavin Herbert Eye Institute, University of California Irvine, Irvine, CA 92697 USA; 8 School of Medicine, Vita-Salute San Raffaele University, Milan, Italy; 9 Division of Head and Neck, Ophthalmology Unit, IRCCS San Raffaele Scientific Institute, Milan, Italy; 10 Centre de Recherche des Cordeliers, From Physiopathology of Retinal Diseases to Clinical Advances, Université de Paris Cité, Inserm, Paris, 75006 France; 11 Department of Ophthalmology, Assistance Publique-Hôpitaux de Paris, Hôpital Cochin, Ophtalmopôle, Paris, 75014 France; 12 Department of Medical Retina and Vitreoretinal Surgery, University of Pittsburgh School of Medicine, 203 Lothrop Street, Suite 800, Pittsburg, PA 15213 USA

**Keywords:** Eplerenone, Central serous chorioretinopathy, Real-world patterns, Treatment, Side-effects

## Abstract

**Purpose:**

To report eplerenone use by retina specialists worldwide for central serous chorioretinopathy (CSCR).

**Methods:**

A self-reporting questionnaire was distributed to retina specialists worldwide to gather clinicians’ perspectives on CSCR cases treated, eplerenone dosage and duration, reasons to use it, and side effects.

**Results:**

The survey included 241 retina specialists (122 Indian and 119 international) with an average experience of 15.69 ± 9.59 years. Oral eplerenone was used to treat CSCR by 149 (62%) participants. Only 6% (n = 9) had easy access to verteporfin dye and photodynamic therapy. 30 (20%) of the 149 respondents changed their treatment with eplerenone after VICI trial results. Eplerenone was prescribed mostly for chronic CSCR (n = 86, 58%), regardless of involvement laterality. 62% (n = 92) had fewer than 25% CSCR cases treated with eplerenone. 85 (57%) respondents used eplerenone only when other treatments failed, while 36 (24%) used it as first-line treatment. 73 (49%) respondents, prescribed eplerenone at a 50 mg daily dose and 137 (92%) retina specialists used eplerenone for 0–3 months. The drug’s efficacy dissatisfied 21 (14%) study participants. 124 (83%) study participants did not encounter any ocular or systemic side effects with eplerenone use. Eplerenone related kidney and electrolyte issues were noted by 11 (7%) study participants.

**Conclusion:**

The treatment of CSCR varies around the world and is primarily influenced by the photodynamic therapy availability and the findings of VICI trial. Despite the limited benefit of eplerenone reported by the VICI trial, it is still used as evidenced by real-world experience.

**Trial Registration Number:**

Not applicable.

**Supplementary Information:**

The online version contains supplementary material available at 10.1186/s40942-023-00500-w.

## Introduction

Central serous chorioretinopathy (CSCR) is a disease with a multifactorial etiology, a complex pathogenesis, and extensive systemic associations that is still poorly understood. It is characterized by fluid accumulation in the neurosensory retinal space, typically in the macula. The subretinal fluid (SRF) in CSCR results from a mismatch between the increased leakage from choroidal vessels, particularly those in Haller’s layer of the choroid, and the decreased SRF absorption due to a dysfunctional retinal pigment epithelium (RPE) [1]. To prevent irreversible RPE damage and permanent visual impairment, the primary objective of CSCR treatment is to achieve faster SRF resolution [1, 2]. In an effort to address the different factors in CSCR pathogenesis, various therapeutic options alone or in combination are being considered, including observation with lifestyle modifications to reduce cortisol levels, pharmacotherapeutic agents, focal thermal laser photocoagulation to the leak, subthreshold micro pulse laser to the region of dysfunctional RPE, and photodynamic therapy (PDT) or transpupillary thermotherapy to the hyperpermeable choroidal vessels and RPE [2].

PDT, which is considered as a mainstay of treatment options in the treatment of CSCR, is neither readily available nor affordable to patients in many parts of the world [3]. This has had an effect on the treatment practices of retina specialists worldwide. In recent years, eplerenone and spironolactone have been the most frequently prescribed pharmacotherapeutic drugs for the treatment of CSCR [4]. Eplerenone and spironolactone are potassium-sparing diuretics with an antagonistic action on mineralocorticoid receptors, approved for myocardial infarction to improve heart remodelling and patients survival [5]. It has been proposed in the management of CSCR based on pre-clinical work showing that mineralocorticoid overactivation induced choroidal features that resemble those in CSCR [6–9]. As a more selective antagonist for mineralocorticoid receptors, eplerenone has fewer hormone-related side effects than spironolactone and is therefore more commonly preferred by retinal specialists [10]. Several articles in the medical literature have already demonstrated the efficacy of oral eplerenone therapy in the acute and chronic management of CSCR [11, 12]. However, variable response with eplerenone therapy in the management of CSCR have been noted frequently in clinical practice.

A large, multicentre, randomized, double-blind, parallel-group, placebo-controlled VICI trial was recently published in which 114 patients with chronic CSCR were randomly assigned to receive either eplerenone or placebo [13]. According to the results of this prospective study, the clinical efficacy of eplerenone was not superior to placebo in patients with chronic CSCR. The requirement per protocol to discontinue treatment in cases of complete SRF resolution or if hyperkalaemia developed was one of the major limitations of this prospective trial. These limitations may have diminished the treatment effect observed as it was clearly shown in the first controlled cross over study analysing the effect of eplerenone, that recurrence occurred after brutal arrest of the treatment [14]. In addition, when prescribed for other indications, mineralocorticoid antagonists are disease modifier drugs, prescribed for a long duration and even a lifetime [15].

Multiple reports from the SPECTRA trial, a randomized, multicentre, open-label study comparing the efficacy and long-term visual outcomes of chronic CSCR patients treated with eplerenone or half-dose PDT, demonstrated the efficacy of the latter in achieving SRF resolution and favourable long-term visual outcomes [16–18]. However, with limited treatment options, inaccessibility to PDT, and CSCR being a multifactorial and difficult-to-treat disease, clinicians around the world continue to use oral eplerenone to treat cases of CSCR [12, 19–22]. Currently, the literature lacks any information regarding practice pattern of eplerenone use among retina specialists for CSCR. In addition, clinicians’ perspectives on the type of CSCR cases treated, the dosage and duration of treatment, reasons to use eplerenone and the side effects of eplerenone would be of great assistance in comprehending the reasons for the contradictory results observed when eplerenone was used to treat CSCR in a real-world setting.

To answer these questions, a web-based questionnaire survey was designed to collect information from retina specialists from around the world regarding their practice patterns with the use of eplerenone in the management of CSCR.

## Methods

The institutional review board of the organization granted the necessary approval for this study’s compliance with ethical standards. This international study utilized a 17-item web-based questionnaire derived from clinical practices associated with the diagnosis and management of CSCR. Before finalizing the questionnaire, it was given to five experienced retina specialists from institutes across the globe and revised based on their feedback. The questionnaire included multiple choice questions as well as short text available to describe specific points if respondent needed. The participants in this survey remained completely anonymous. Questionnaire has been added as supplement (Supplement 1). The questionnaire included basic information about the participants, such as their years of retina practice experience and the country in which they conducted their clinical practice. In addition, the survey inquired about the number of CSCR cases observed per month as well as the preferred treatment options considered for the various clinical presentations of CSCR. The survey also included questions regarding the utilization of oral eplerenone in the treatment of CSCR by retina specialists and also their experiences related to the side-effects of the drug. All respondents were allowed to submit the survey only once.

### Statistical analysis

All data were analysed using GraphPad Prism version 9.5.1 (733) for Windows, (GraphPad Software, San Diego, California USA. www.graphpad.com). Quantitative variables between the 2 groups were analysed using the Mann-Whitney U test. Chi-square test was used to compare the categorical data between the 2 groups. P values < 0.05 were considered statistically significant.

## Results

A total of 241 retina specialists from around the world participated in the survey. There were 122 (51%) Indian participants and 119 (49%) participants from other regions of the world (North America – 28; South America – 8; Asia other than India – 35; Africa – 1; Pacific − 3; Europe – 44). These participants’ average years of experience were 15.69 ± 9.59 (range: 2–45 years). According to the responses of the participants, the majority of retina specialists identified fewer than 10 CSCR new patients per month (n = 157, 65%), including both acute (n = 229, 95%) and chronic (n = 202, 84%) cases. In this survey, 92 (38%) study participants employed PDT as a treatment option. 149 (62%) survey respondents had experience using oral eplerenone for CSCR management. Observation and lifestyle modifications (n = 215, 89%), thermal focal laser (n = 140, 58%), subthreshold micro pulse laser (n = 80, 33%), and anti-vascular endothelial growth factor (n = 61, 25%) therapy were additional treatment options for CSCR management utilized by the study participants in their retina practices. For 136 (56%) participants in the study, PDT was unavailable. While PDT was available but difficult to acquire the drug for 75 (31%) of the study participants, it was readily accessible for the remaining 30 (13%) participants (Table 1).

**Table 1:** Responses from retina specialists having experience with CSCR management:

Variable		Value
Total Number of responses		241
Years of clinical experience (Mean ± SD)		15.69 ± 9.589
Geographic location (n, %)	India	122 (51)
	Rest	119 (49)
	North America	28 (12)
	South America	8 (3)
	Asia (other than India)	35 (14)
	Europe	44 (18)
	Africa	1 (1)
	Pacific	3 (1)
No. of new patients with CSCR seen in a month (n, %)	0–10	157 (65)
	11–20	62 (26)
	> 20	22 (9)
Acute cases of CSCR seen in a month (n, %)	0–10	229 (95)
	11–20	9 (4)
	> 20	3 (1)
Chronic cases of CSCR seen in a month (n, %)	0–10	202 (84)
	11–20	33 (14)
	> 20	6 (2)
Treatment modalities used in CSCR (n, %)	Observation	215 (89)
	PDT	92 (38)
	Focal laser	140 (58)
	Subthreshold MP laser	80 (33)
	AntiVEGF	61 (25)
	Eplerenone	149 (62)
	Others	32 (13)
Availability of PDT in practice (n, %)	No, not available	136 (56)
	Yes, but difficult to get the drug	75 (31)
	Yes, easily available	30 (13)

Abbreviations: CSCR – central serous chorioretinopathy; PDT – photodynamic therapy; MP – micro pulse; VEGF – vascular endothelial growth factor; SD – standard deviation

A total of 149 (62%) study participants with prior experience with oral eplerenone therapy had their responses to questions regarding the drug’s application, dosage, and adverse effects evaluated. Only nine (6%) retina specialists out of these 149 study participants had easy access and availability to verteporfin dye and PDT. On enquiring the participants if the results of the VICI trial had altered their treatment practice with eplerenone, 30 (20%) of the 149 respondents indicated that the results had altered their practice, while 70 (47%) of the respondents did not believe that the results had influenced their practice. According to the majority of survey respondents (n = 86), eplerenone was prescribed primarily for chronic CSCR cases and involvement laterality had no bearing on eplerenone treatment. 62% (n = 92) of the respondents had < 25% of CSCR cases treated with eplerenone. According to 85 (57%) respondents, eplerenone was only used when other treatment methods failed, and only 36 of the 149 respondents used it as their first line of therapy. In this survey, 73 (49%) respondents prescribed a daily dose of 50 mg of eplerenone. Only 19 (13%) respondents prescribed a daily dose of 25 mg. According to 137 retina specialists, treatment duration ranged from 0 to 3 months. Only 21 participants in the study were either dissatisfied or completely dissatisfied with the drug’s effectiveness. 124 of the 149 study participants did not experience any ocular or systemic adverse effects related to eplerenone. The minority of study participants (n = 11) reported adverse kidney and electrolyte effects following eplerenone administration (Table 2).

**Table 2:** Responses from retina specialists having experience with CSCR management and eplerenone therapy:

Retinal specialists having experience with eplerenone therapy (n)		149
Availability of PDT in practice (n, %)	No, not available	94 (63)
	Yes, but difficult to get the drug	46 (31)
	Yes, easily available	9 (6)
Effect of VICI trial results on eplerenone usage (n, %)	Yes	30 (20)
	No	70 (47)
	Somewhat	49 (33)
Indication for Eplerenone use (n, %)	Acute	22 (15)
	Chronic	86 (58)
	Both	41 (27)
Laterality consideration for Eplerenone use (n, %)	Unilateral	6 (4)
	Bilateral	8 (5)
	Doesn’t matter	135 (91)
% of patients are/were on eplerenone treatment (n, %)	0–25	92 (62)
	25–50	33 (22)
	50–75	12 (8)
	75–100	12 (8)
Prescription of Eplerenone in the current practice (n, %)	Yes routinely	50 (34)
	Only when there is no response to available options	85 (57)
	Prescribed earlier, not now	14 (9)
When to prescribe Eplerenone (n, %)	First line	36 (24)
	Second line as monotherapy	63 (42)
	Third line as combination	17 (11)
	Desperate situations	33 (22)
Daily Dose of Eplerenone (n, %)	25	19 (13)
	50	73 (49)
	25–50	57 (38)
Duration of Eplerenone use (n, %)	0–3	137 (92)
	4–6	10 (7)
	> 6	2 (1)
Satisfaction levels with eplerenone (n, %)	Very satisfied	6 (4)
	Satisfied	67 (45)
	Neutral	55 (37)
	Dissatisfied	16 (11)
	Very dissatisfied	5 (3)
Side effects (n, %)	Renal issues	11 (7)
	Gastro-intestinal disturbances	3 (2)
	Cardiac problem	4 (3)
	Muscle pain	2 (1)
	Generalised weakness	3 (2)
	Others	2 (1)
	Not experienced	124 (83)

Abbreviations: CSCR – central serous chorioretinopathy; PDT – photodynamic therapy

### Analysis

In this survey, a significantly higher proportion of Indian retina specialists than those from the rest of the world (India: 95/122, Rest of the World: 54/119, p < 0.001) had clinical experience with the use of oral eplerenone for the treatment of CSCR. Table 3 compares the responses of retina specialists from India and the rest of the world regarding the usage, application, dosage, and adverse effects of the drug. We observed that the use of oral eplerenone for the management of CSCR varied significantly between the two geographical groups, based on variables such as the availability of PDT and the results of the VICI trial (p < 0.05). On the other hand, a significant proportion of retina specialists from India had experience of treatment with eplerenone only in chronic cases of CSCR (p < 0.05). The use of eplerenone and the timing of its use varied significantly among retinal specialists from around the world (p < 0.05). Between the two groups, there was a significant difference in the prescribed daily dosage of the drug. Compared to other retinal specialists who used a higher daily dose of 50 mg, many Indian retinal specialists administered 25 mg of eplerenone daily (p < 0.05). In CSCR, the levels of satisfaction with outcomes following the use of eplerenone are significantly different between the two groups. The Indian retinal specialists appeared more satisfied with the use of eplerenone than retinal specialists from other countries (p < 0.05). Both groups of retinal specialists who used eplerenone experienced comparable adverse reactions to the drug (p > 0.05).

**Table 3:** Comparing the experiences between retina specialists from India and other parts of the globe:

Variable		India (n = 95)	Rest of the World (n = 54)	P value
Years of clinical experience		13.96 ± 9.17	17.44 ± 9.307	0.019
No. of new patients with CSCR seen in a month (n, %)	0–10	61 (64)	34 (63)	0.861
	11–20	26 (27)	14 (26)	
	> 20	8 (9)	6 (11)	
Acute cases of CSCR seen in a month (n, %)	0–10	87 (92)	52 (96)	0.072
	11–20	7 (7)	0 (0)	
	> 20	1 (1)	2 (4)	
Chronic cases of CSCR seen in a month (n, %)	0–10	88 (93)	37 (69)	0.001
	11–20	7 (7)	13 (24)	
	> 20	0 (0)	4 (7)	
Availability of PDT in practice (n, %)	No, not available	76 (80)	18 (33)	< 0.001
	Yes, but difficult to get the drug	18 (19)	28 (52)	
	Yes, easily available	1 (1)	8 (15)	
Effect of VICI trial results on eplerenone usage (n, %)	Yes	14 (15)	16 (30)	0.011
	No	53 (56)	17 (31)	
	Somewhat	28 (29)	21 (39)	
Indication for Eplerenone use (n, %)	Acute	12 (13)	10 (19)	0.045
	Chronic	62 (65)	24 (44)	
	Both	21 (22)	20 (37)	
Laterality consideration for Eplerenone use (n, %)	Unilateral	2 (2)	4 (7)	0.189
	Bilateral	4 (4)	4 (7)	
	Doesn’t matter	89 (94)	46 (85)	
% of patients are/were on eplerenone treatment (n, %)	0–25	59 (62)	33 (61)	0.812
	25–50	20 (21)	13 (24)	
	50–75	7 (7)	5 (9)	
	75–100	9 (10)	3 (6)	
Prescription of Eplerenone in the current practice (n, %)	Yes routinely	36 (38)	14 (26)	0.011
	Only when there is no response to available options	55 (58)	30 (56)	
	Prescribed earlier, not now	4 (4)	10 (18)	
When to prescribe Eplerenone (n, %)	First line	20 (21)	16 (30)	0.501
	s line as monotherapy	40 (42)	23 (43)	
	Third line as combination	13 (14)	4 (7)	
	Desperate situations	22 (23)	11 (20)	
Daily Dose of Eplerenone (n, %)	25	18 (19)	1 (2)	0.01
	50	44 (46)	29 (54)	
	25–50	33 (35)	24 (44)	
Duration of Eplerenone use (n, %)	0–3	86 (91)	51 (94)	0.506
	4–6	7 (7)	3 (6)	
	> 6	2 (2)	0 (0)	
Satisfaction levels with eplerenone (n, %)	Very satisfied	5 (5)	1 (2)	0.002
	Satisfied	50 (53)	17 (31)	
	Neutral	34 (36)	21 (39)	
	Dissatisfied	4 (4)	12 (22)	
	Very dissatisfied	2 (2)	3 (6)	
Side effects (n, %)	Renal issues	4 (4)	7 (13)	0.325
	GI disturbances	2 (2)	1 (2)	
	Cardiac problem	2 (2)	2 (4)	
	Muscle pain	1 (1)	1 (2)	
	Generalised weakness	1 (1)	2 (4)	
	Others	2 (2)	0 (0)	
	Not experienced	83 (88)	41 (72)	

Abbreviations: CSCR – central serous chorioretinopathy; PDT – photodynamic therapy

## Discussion

This survey compiled the experiences of retina specialists from around the world and India regarding CSCR management including the use of eplerenone. The survey revealed that approximately 62% of retina specialists were familiar with oral eplerenone therapy for the treatment of CSCR. In terms of eplerenone usage, preferred cases, preferred time for treating, dosage and duration of treatment, satisfaction with treatment, and eplerenone side effects, we observed significant differences between retina specialists from India and those from other regions of the world. The survey revealed that CSCR is a common retinal pathology, with more than 50% of respondents observing up to ten new cases per month, including both acute and chronic forms of the disease. The average experience of survey respondents was approximately 15 years, making the survey’s findings credible, significant, and pertinent.

In ophthalmology, PDT with verteporfin has a wide range of indications, including central serous chorioretinopathy [3, 23, 24]. PDT is an effective treatment for CSCR regarding the rate of SRF reabsorption, particularly in cases of acute CSCR with sub/juxta foveal leaks, recurrent CSCR, and chronic CSCR [23, 24]. Due to its wide applicability and efficacy in different stages of CSCR, PDT is either the primary treatment or considered a major treatment option in the management of CSCR, although its exact mechanisms of action remains unclear [1, 3]. Particularly, the long term effect of repeated PDT on RPE cells from complex CSCR [25] remains to be evaluated since verteporfin PDT, even when used at half fluence or half dose was shown to cause histological damages to RPE cells [26, 27].

Since July 2021, there has been a global shortage of verteporfin (Visudyne®), an essential drug required for PDT. The shortage of verteporfin has had a significant impact on the care of ophthalmic patients around the world, and may have resulted in significant and irreversible vision loss, as well as the need to consider alternatives to PDT [28]. In our survey, we found that only 12% of retina specialists were able to access Visudyne® and perform PDT on CSCR patients without difficulty. 63% of the 149 retinal specialists surveyed who had experience using eplerenone did not have access to photodynamic therapy (PDT), and another 30% found it difficult to obtain the drug. Only 7% of respondents who were using eplerenone also had easy access to PDT. In comparison to respondents from the rest of the world, those from India had more experience using eplerenone in CSCR management and had no access to verteporfin and PDT. Thus, the non-availability of PDT in countries such as India may have compelled clinicians to seek alternative therapy for CSCR management, and eplerenone has emerged as a viable alternative to PDT for CSCR management.

An important and exciting finding from this survey was that approximately 58% (86/149) of respondents used eplerenone in chronic cases of CSCR, while only 15% used it in acute cases of CSCR. The acute form of CSCR is caused by increased choroidal vascular permeability, which results in focal changes in the RPE, such as RPE detachment, followed by a micro RPE rip and fluid accumulation in the neurosensory space. In contrast, the chronic form of CSCR is characterized by diffuse rather than focal RPE abnormalities, resulting in dysfunctional RPE and persistent subretinal fluid [29]. During systemic corticosteroid treatment, RPE and choriocapillaris receptor reactivity may reveal the strong association between steroid use and CSCR [30]. Daruich et al. hypothesized that excessive activation of the mineralocorticoid receptor in the choroidal endothelial cells by aldosterone or glucocorticoids induces upregulation of the vasodilator potassium channel KCa2.3, which modulates smooth muscle relaxation in the choroidal vessels. In addition, mineralocorticoid receptor activation in human RPE cells derived from induced pluripotent stem cells showed deregulation of genes involved in RPE barrier integrity, transcellular transport and choroid functions [8], supporting the use of mineralocorticoid antagonists in the treatment of acute CSCR [31]. Similarly, a recent study published by our group on the effects of oral eplerenone therapy in acute cases of CSCR found faster SRF resolution, rapid improvement in visual acuity, fewer recurrences in the affected eye, a lower risk of developing CSCR in the contralateral eye, and fewer ocular and systemic side effects [12]. The addition of oral eplerenone therapy to lifestyle modifications in acute cases of CSCR could expedite the resolution of SRF, prevent long-term RPE damage, and improve visual outcomes.

Animal models in which mineralocorticoid receptor is activated either by chronic aldosterone exposure or by overexpression of the human receptor show a pachychoroid-like phenotype, suggesting that mineralocorticoid pathway activation may favour choroidal pathology [7] which may indicate that MR antagonists could have a long term effect on the pachychoroid phenotype, which has not been yet evaluated. Indeed, all studies on CSCR evaluate as the main endpoint, the resolution of subretinal fluid, and not the underlying mechanisms causing this sign.

A large-scale, multicentre, randomised VICI trial evaluating the efficacy of eplerenone in patients with persistent CSCR failed to demonstrate a superior beneficial response to placebo [13]. The VICI trial was a randomized, double-blind, multicentre, placebo-controlled study from the United Kingdom that was designed to assess the efficacy of eplerenone in the treatment of active, previously untreated CSCR for more than four months [13]. Patients were administered eplerenone or a placebo (when fluid was present) for 12 months. Improvement in visual acuity was the primary outcome measure. The results of the study suggested that eplerenone therapy was not superior to placebo in the treatment of persistent CSCR. According to the findings of this survey, the results of the VICI trial did influence the treatment practices of retina specialists in the management of CSCR. 44% (42/95) of retina specialists from India and 69% (37/54) of retina specialists from the rest of the world confirmed in the survey that the results of the VICI trial had an impact on their CSCR management practices. This observation was supported by statistical evidence. The absence of PDT in clinical practice in India may have been the most likely explanation for why the VICI trial results had less of an impact on Indian retinal specialists than on their counterparts in the rest of the world. Consequently, Indian retina specialists view oral eplerenone as a viable alternative to PDT.

All respondents to the current survey administered eplerenone to CSCR patients at starting doses ranging from 25 to 50 mg per day and for a variable period of time. Even in the recently published prospective VICI trial, eplerenone therapy was discontinued upon complete resolution of SRF or if the patient’s potassium levels increased. There was no established dosage protocol for eplerenone use in CSCR. But in other diseases in which eplerenone or other MR antagonists are approved, the drugs were shown to act of tissue remodelling and play a role as disease modifying drugs.

Compared to retina specialists from other countries, retina specialists from India used a much lower starting dose of 25 mg per day. In cases of CSCR, a lower starting dose of eplerenone may have been administered out of concern for systemic side effects. However, there were no significant differences between the side effect profiles reported by Indian retina specialists and their counterparts from the rest of the world after CSCR treatment with eplerenone. The levels of treatment satisfaction among retinal specialists from the two distinct geographic regions were also significantly distinct. Compared to their Indian counterparts, retinal specialists from countries other than India experience poor satisfaction levels following eplerenone treatment.

One of the most significant limitations is the size of the survey’s sample. The number of retina specialists who participated in this survey was disproportionately small compared to the total number of retina specialists in the world. A minimum sample size was required to ensure the validity of the study and analysis. The primary advantage of this paper is that it provides first-hand accounts of the observations made by clinicians following the use of eplerenone in the management of CSCR.

In conclusion, the treatment of CSCR varies across the globe and is primarily influenced by the availability of PDT and the results of an established clinical trial such as the VICI trial. In spite of limited benefit of eplerenone reported by VICI trial, eplerenone use is continued as supported by real-life experience.

### Electronic supplementary material

Below is the link to the electronic supplementary material.

Supplementary Material 1

## Data Availability

The datasets used and/or analysed during the current study are available from the corresponding author on reasonable request.

## Declarations

### Animal research

“This article does not contain any studies with animals performed by any of the authors.”

## Abbreviations

CSCR: Central serous chorioretinopathy
SRF: Subretinal fluid
RPE: Retinal pigment epithelium
PDT: Photodynamic therapy
