# Supplementary material for: Real-world practice patterns of eplerenone use for central serous chorioretinopathy
Source: Int J Retina Vitreous. 2023 Oct 2;9:61. doi: 10.1186/s40942-023-00500-w (PMC10544617; doi:10.1186/s40942-023-00500-w)
Supplement: Supplementary file 1 — Supplementary Material 1 [file 40942_2023_500_MOESM1_ESM.docx]

***Supplement 1: Questionnaire survey regarding the use of eplerenone for central serous chorioretinopathy (CSCR) in real-life practice patterns***

1. Number of years in clinical practice (years) -
2. Country of practice – please specify
3. How many new patients with CSCR do you see in a month?
   1. 0-10
   2. 11-20
   3. > 20
4. Among the CSCR patients you manage in a month, how many are acute cases?
   1. 0-10
   2. 11-20
   3. > 20
5. Among the CSCR patients you manage in a month, how many are chronic cases?
   1. 0-10
   2. 11-20
   3. > 20
6. Which modalities do you use for CSCR (both acute/chronic) management (check multiple options)?
   1. Observation
   2. Photodynamic therapy
   3. Focal laser
   4. Sub threshold micro pulse laser
   5. Anti VEGF
   6. Eplerenone
   7. Others (please specify)
7. What is the availability of PDT in your practice?
   1. Yes, easily available
   2. Yes, but difficult to get a drug
   3. No, not available at all
8. Did VICI trial results (trial reported eplerenone has no benefit over placebo) change your Eplerenone usage in clinical practice?
   1. Yes
   2. No
   3. Somewhat
9. If you use eplerenone, what are your indications?
   1. Acute cases
   2. Chronic cases
   3. Both
10. If you use eplerenone, what are your indications?
    1. Unilateral cases
    2. Bilateral cases
    3. Both
    4. Laterality doesn’t matter
11. If you use eplerenone, what percentage of your CSCR patients are/were on eplerenone?
    1. 0-25%
    2. 25-50%
    3. 50-75%
    4. 75-100%
12. Do you currently prescribe Eplerenone in the management of CSCR?
    1. Yes, routinely
    2. Only when there is no response to available options
    3. Prescribed earlier, but now I don’t prescribe
    4. Never prescribed
13. If you use eplerenone, at what stage do you prescribe?
    1. First line
    2. Second line as monotherapy
    3. Third line as combination therapy
    4. Only in desperate situation when there is no response to any treatment
14. What is the treatment protocol do you follow? – please specify the dose/duration
15. What has been your overall satisfaction level with Oral Eplerenone?
    1. Very satisfied
    2. Satisfied
    3. Neutral
    4. Dissatisfied
    5. Very dissatisfied
16. Any specific side effects if you experienced with Eplerenone – please specify
17. Any comments – please specify
